# On the Temperature Behavior of Pulse Propagation and Relaxation in Worms, Nerves and Gels

**DOI:** 10.1371/journal.pone.0066773

**Published:** 2013-06-21

**Authors:** Christian Fillafer, Matthias F. Schneider

**Affiliations:** Department of Mechanical Engineering, Boston University, Biological Physics Group, Boston, Massachusetts, United States of America; Jacobs University Bremen, Germany

## Abstract

The effect of temperature on pulse propagation in biological systems has been an important field of research. Environmental temperature not only affects a host of physiological processes *e.g.* in poikilotherms but also provides an experimental means to investigate the thermodynamic phenomenology of nerves and muscle. In the present work, the temperature dependence of blood vessel pulsation velocity and frequency was studied in the annelid *Lumbriculus variegatus*. The pulse velocity was found to vary linearily between 0°C and 30°C. In contrast, the pulse frequency increased non-linearly in the same temperature range. A heat block ultimately resulted in complete cessation of vessel pulsations at 37.2±2.7°C (lowest: 33°C, highest: 43°C). However, quick cooling of the animal led to restoration of regularly propagating pulses. This experimentally observed phenomenology of pulse propagation and frequency is interpreted without any assumptions about molecules in the excitable membrane (*e.g.* ion channels) or their temperature-dependent behaviour. By following Einstein’s approach to thermodynamics and diffusion, a relation between relaxation time τ and compressibility κ of the excitable medium is derived that can be tested experimentally (for κ_T_ ∼ κ_S_). Without fitting parameters this theory predicts the temperature dependence of the limiting (*i.e.* highest) pulse frequency in good agreement with experimental data. The thermodynamic approach presented herein is neither limited to temperature nor to worms nor to living systems. It describes the coupling between pulse propagation and relaxation equally well in nerves and gels. The inherent consistency and universality of the concept underline its potential to explain the dependence of pulse propagation and relaxation on any thermodynamic observable.

## Introduction

In typical studies of propagative phenomena in single cells and tissue, the experimenter deliberately induces pulses in an otherwise quiescent, resting system. During most physiological processes, however, pulses are generated in a recurring manner because of, for instance, a persisting stimulus or a system-inherent pacemaker mechanism that generates auto-excitations. It is of fundamental interest to understand the mechanisms that underlie these processes.

**Figure 1:**
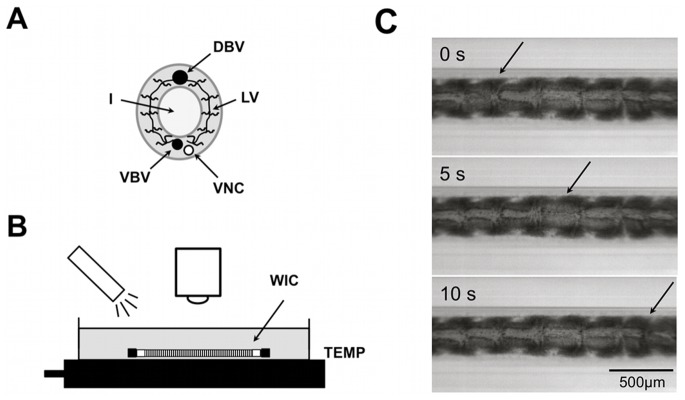
Study of pulse wave propagation in *Lumbriculus variegatus*. (**A**) Cross-sectional view of a *Lumbriculus* segment with intestine (I), ventral nerve chord (VNC), ventral (VBV) and dorsal blood vessel (DBV). The latter are partially connected by lateral vessels (LV). (**B**) A blackworm is aspirated into a buffer-filled glass capillary (WIC) and subsequently submersed in a temperature-controlled petri dish (TEMP). (**C**) Top view of WIC with the DBV (light-gray structure in the center) and a propagating pulse wave (arrow).

The present work is concerned with the effect of environmental temperature on blood vessel pulsations in the poikilotherm *Lumbriculus variegatus*. *Lumbriculus* is a small annelid with a body that is widely transparent and thus well suited for observations of physiological processes within. The dorsal blood vessel of the animal is a contractile, muscular tube, which phyllogenetically represents a predecessor of the hearts of higher organisms (Fig. 1). From an experimentalist’s point of view, blackworms are an excellent model system to study basic principles of rhythmical excitation processes [1]. The propagation velocity of vessel contractions as well as the pulse frequency in the dorsal vessel can be extracted readily by light microscopy. Studies of the effect of temperature on these parameters are of twofold interest. First, they can provide insight into how bodily functions of poikilotherms are affected by as well as adapted to *e.g.* diurnal or annual variations of environmental temperature. Second, temperature represents a thermodynamic state variable that can be controlled comparatively well in experiments with whole animals. Thus, such studies can facilitate an understanding of excitable cells and tissues within the theoretical framework of thermodynamics.

It is the particular aim of the present work to elaborate on the latter. To establish a solid experimental basis for further discussions, the propagation velocity as well as frequency of blood vessel pulsations in *Lumbriculus* will be determined as functions of temperature. These data will be interpreted in the framework of a thermodynamic theory. It has been suggested in the past [2], [3] and more recently [4], [5] that excitation processes in biological systems could be based on acoustic waves. Strong support for such a view is lent by the macroscopic thermal [6]–[8], mechanical [9]–[11], optical [12]–[14] *and* electrical phenomenology [14], [15] of action potentials in single cells as well as spreading depression waves in nervous tissue. Thermodynamically, the assumption of propagating acoustic waves leads to testable predictions about the temperature-dependence of the macroscopic mechanical material properties of the excitable medium and its inherent relaxation. It will be shown that a thermodynamic relation between compressibility (κ) and relaxation time (τ) can be used to predict the temperature-dependent pulse frequency of blackworms correctly. Finally, the same concept will be applied to interpret data from the literature on propagating (chemical) waves in gels [16] and action potentials in human nerves [17]. The general agreement between theory and experiment across living and non-living systems underlines the consistency and integrative nature of the presented thermodynamic approach.

## Materials and Methods

### 1.1 *Lumbriculus variegatus*

*Lumbriculus variegatus* was obtained from Carolina Biological (Burlington, NC, USA). The worms were cultivated in spring water (Poland Spring; Poland, ME, USA) at 9–11°C for at least four weeks prior to experiments. Studies with *Lumbriculus variegatus* do not require approval by the institutional animal care committee.

### 1.2 Setup to Study Blood Vessel Pulsations

After 20 minutes of pre-incubation in spring water buffered to pH 7.0 with 5 mM HEPES/NaOH, a worm was gently aspirated into a glass capillary (20 µL; Drummond Scientific Company, Broomall, PA, USA). This step had to be done with particular care in order to avoid injury to the animal. The capillary was subsequently trimmed with a glass cutter to the worm’s body length and capped with rubber plugs. The worm-filled capillary was immersed in a petri dish whose temperature was controlled by a subjacent Peltier element (Fig. 1B). The dorsal blood vessel and pulsations therein are clearly observable by light microscopy (Fig. 1C). After every change of temperature a worm was left to equilibrate for 2–3 minutes. This period was typically sufficient for the pulse frequency to stabilize. Vessel pulsations at the tail end of the worm are generally more frequent and irregular than in the mid-body and head regions [1]. In order to ensure comparability, segments in the mid-body region of the worm were studied. The number of pulse waves travelling through an arbitrarily chosen segment was counted for one minute. This procedure was repeated six times at each temperature level and from the collected data an average pulse frequency was obtained. Generally, 3–4 temperature levels were studied per worm within about 45 minutes. Worms were not reused in later experiments.

The pulse propagation velocity was obtained as follows: The projected length of the vessel within the optical field of view was determined by manual delineation of the vessel edge in ImageJ (NIH, Bethesday, MA, USA). The typical projected vessel length was ∼3.3 mm. The propagation velocity was calculated by dividing this length by the time that it took for a pulse to cross it.

## Results and Discussion

### 2.1 Effect of Temperature on Blood Vessel Pulsation

#### Pulse propagation velocity

**Figure 2:**
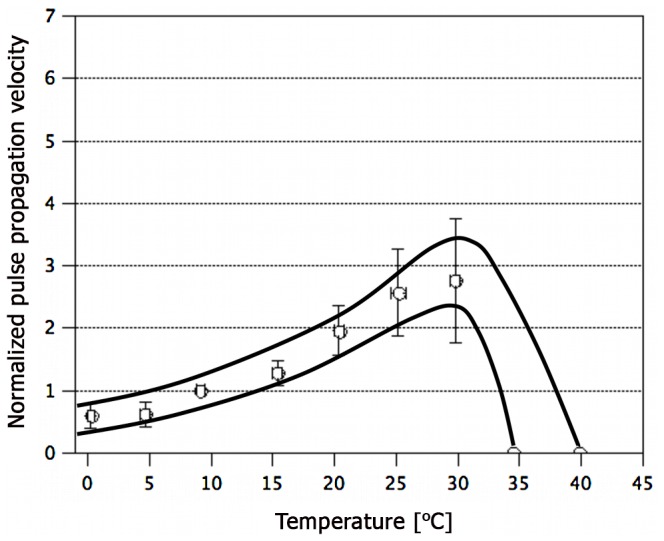
Variation of pulse wave velocity with environmental temperature. Data was normalized to each individual worm’s pulse propagation velocity at 9.2±0.3°C (average: 0.19±0.05 mm s^−1^). The black envelopes are guides to the eye. Each data point represents the average of at least 36 measurements. Number of worms studied = 29.

When blackworms were exposed to different environmental temperatures, a characteristic variation of the propagation velocity *c* of blood vessel pulsations in the dorsal vessel was observed (Fig. 2). It was found that *c* varies linearily with temperature in the range between 0°C and 30°C. At 9.2±0.3°C the average pulse wave velocity was 0.19±0.05 mm s^−1^. Since the worms had been adapted to an environmental temperature of 9–11°C for four weeks prior to the measurements, this value will be denoted as the “basal velocity”. It is important to bear in mind that the pulse velocities discussed herein were calculated based on the projected length of the blood vessel as extracted by microscopy. The “real” distance that a pulse covers in the excitable medium could certainly be much larger. For example, folds at the cell (plasma membrane) and tissue level (blood vessel surface) might lead to a considerable difference between actual path length and projected vessel length. However, unless the ratio between projected length and actual path length varies strongly with temperature, it is to be expected that the temperature-dependence in Fig. 2 will be conserved and that the real velocities are simply offset by a constant.

**Figure 3:**
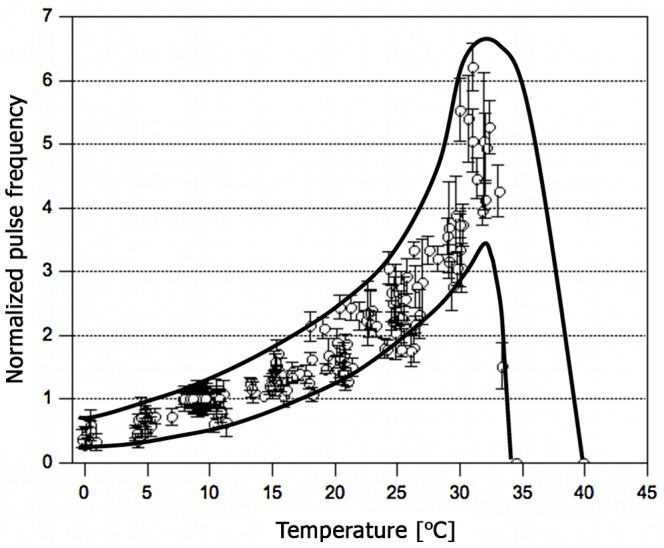
Variation of pulse frequency with environmental temperature. Data was normalized to each individual worm’s pulse frequency at 9.3±0.7°C (average: 4.4±0.8 beats min^−1^). The black envelopes are guides to the eye. Each data point represents the average of six measurements. Number of worms studied = 68.

At the lowest temperature tested (∼0°C), *c* was reduced to about half of the basal velocity while at 20°C and 30°C an about 2 and 2.8 fold higher pulse velocity was observed respectively. Above 30°C, blood vessel pulsations became irregular and when the temperature was increased further they ceased altogether as will be discussed below. The relatively higher standard deviation of the velocity data at temperatures ≥20° (Fig. 2) is probably a measurement artifact. A blackworm in a glass capillary remains rather motionless in the temperature range between 0°C and 20°C. However, at higher temperatures motility of the worms - in the form of repeated body reversal, stretching as well as back-and-forth movement - clearly increases. These translocations of the worm lead to irregular changes of the projected vessel length in the field of view and thereby could result in a higher variability of the velocity data. The black envelopes in the temperature range between the last data points and the heat block temperatures (Fig. 2 and Fig. 3) are interpolations. Blood vessel pulsations are highly irregular at these temperatures and thus it was hard to obtain meaningful data.

The velocity-temperature relationship for vessel pulsations in *Lumbriculus* (Fig. 2) follows a pattern that seems to be conserved for many types of pulses in excitable systems: a linear increase of *c* with increasing temperature which is ultimately interrupted by a heat block. Qualitatively identical results have, for instance, been reported for action potentials in single cells such as squid giant axons [18], [19], nerve fibers from frog [20]–[22] as well as cat [21], [23] and humans [24]. Moreover, similar observations have been made for excitation phenomena on the tissue level. The temperature-velocity relationship of Ca^2+^-waves as well as that of spreading depression waves in brain cortex are well studied examples [25], [26]. It also has to be underlined that the discussed phenomenology is not a peculiarity of animal or human organisms. Equivalent results have indeed been obtained for action potential propagation in *Chara* and *Nitella* plant cells by our lab (unpublished data) and by other groups [27], [28]. Finally, even pulses in non-living excitable media such as gel-based Belousov-Zhabotinsky reaction systems are characterized by a very similar temperature-velocity profile [16]. This remarkable conservation of the temperature dependence of the pulse propagation velocity has to be the consequence of a well-conserved physical mechanism.

#### Pulse frequency

It is fundamentally interesting to study if the pulse velocity and frequency of periodic excitations in cells and tissues (*e.g.* heart, intestine, etc.) are related. Thus, in addition to the temperature dependence of *c*, the temperature-frequency dependence of blood vessel pulsations was determined. As illustrated in Fig. 3, the pulse frequency follows a similar qualitative behavior, but with a steeper rise. The deviation from linearity becomes particularly evident between 30–35°C where up to 6-fold higher frequencies as compared to the basal rate (4.4±0.8 beats min^−1^ at 9.3±0.7°C) were observed.

#### Heat-block of blood vessel pulsations

**Figure 4:**
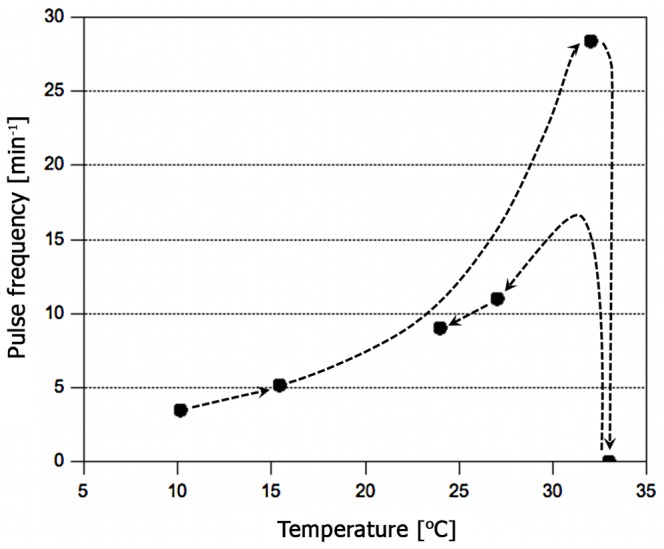
Reversible heat-block of pulse propagation. Heating of a worm above a critical temperature (average threshold temperature: 37.2±2.7°C; min: 33°C, max: 43°C; number of worms studied = 17) led to cessation of blood vessel pulsations. Regular contractions reappeared upon quick cooling. Illustrated is a typical temperature-frequency response as obtained from a single worm. Dashed lines are guides to the eye.

Blood vessel pulsations in blackworms cease altogether if the worm is heated above a critical temperature. The absolute temperature at which such a heat block occurs varies between species and is moreover shifted by adaptation to environmental factors like *e.g.* temperature in poikilotherms [29]. In *Lumbriculus*, cardiac arrest typically sets in at 37.2±2.7°C (lowest: 33°C, highest: 43°C). However, unless this temperature or an even higher one is sustained for several minutes, the worm can be brought back to life by rapid cooling to ∼5°C below the individual critical temperature. Upon subsequent expulsion of the animal from the glass capillary, partial deformations of the body segments were observed in several cases. The severity of such deformations seemed to increase with prolonged exposure of the worm to high temperature. Despite these irreversible effects it is remarkable that in most cases blood vessel pulsations were – with slight hysteresis – restored to the typical frequencies by rapid cooling (Fig. 4). This underlines the reversible component of the heat block phenomenon which has also been observed in other biological systems [18], [19].

### 2.2 Thermodynamic Concept for Pulse Propagation and Predictions

The temperature dependence of the propagation velocity of pulses in nerve and muscle is typically ascribed to temperature sensitivities of molecular components of the excitable membrane (*i.e.* ion channels). However, such molecular interpretations are inherently difficult to test since ion channels presently can not be studied in absence of a membrane which – by itself – has a temperature sensitivity. Furthermore, the similarities between the temperature-velocity relationships for pulses in living [19], [21], [24], [26], [28] and non-living systems [16], indicate that – after all – a more general theory will be necessary to explain the underlying mechanisms.

#### A phenomenological thermodynamic approach

It has been suggested that pulses in biological systems are thermodynamic processes that resemble acoustic waves [2]–[5]. This view is strongly supported by the macroscopic thermal [6]–[8], mechanical [9]–[11], optical [12]–[14] *and* electrical phenomenology [14], [15] of action potentials as well as spreading depression waves. In fact, it has been shown very recently that the propagation velocity *c* of pulses in lipid monolayers – the simplest model of a cell membrane – can be described by an expression similar to the well-established expression for linear sound
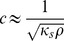
(1)with κ*_S_* as the isentropic mechanical compressibility and ρ as the density of the medium [5], [30]. The compressibility of a 2-dimensional medium (*e.g.* an excitable cell membrane) is a macroscopic material property defined as 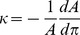 with *A* as the surface area and π as the lateral pressure. It represents the mechanical susceptibility or “softness” of a system. κ, as well as all other macroscopic material properties such as the heat capacity *c_P_*, electrical capacitance *C_T_*, thermal expansion coefficient α*_T_*, etc., depend on the thermodynamic state of the system and thus can be obtained from state diagrams. Any state change, for example by heating/cooling, stretching/compression, exposure to high pH levels or ion concentrations, etc. will lead to the realization of a new set of state diagrams or material properties. It is clear from Eq. (1) that state change-induced variations of κ should be reflected in the velocity of pulses in the system.

**Figure 5:**
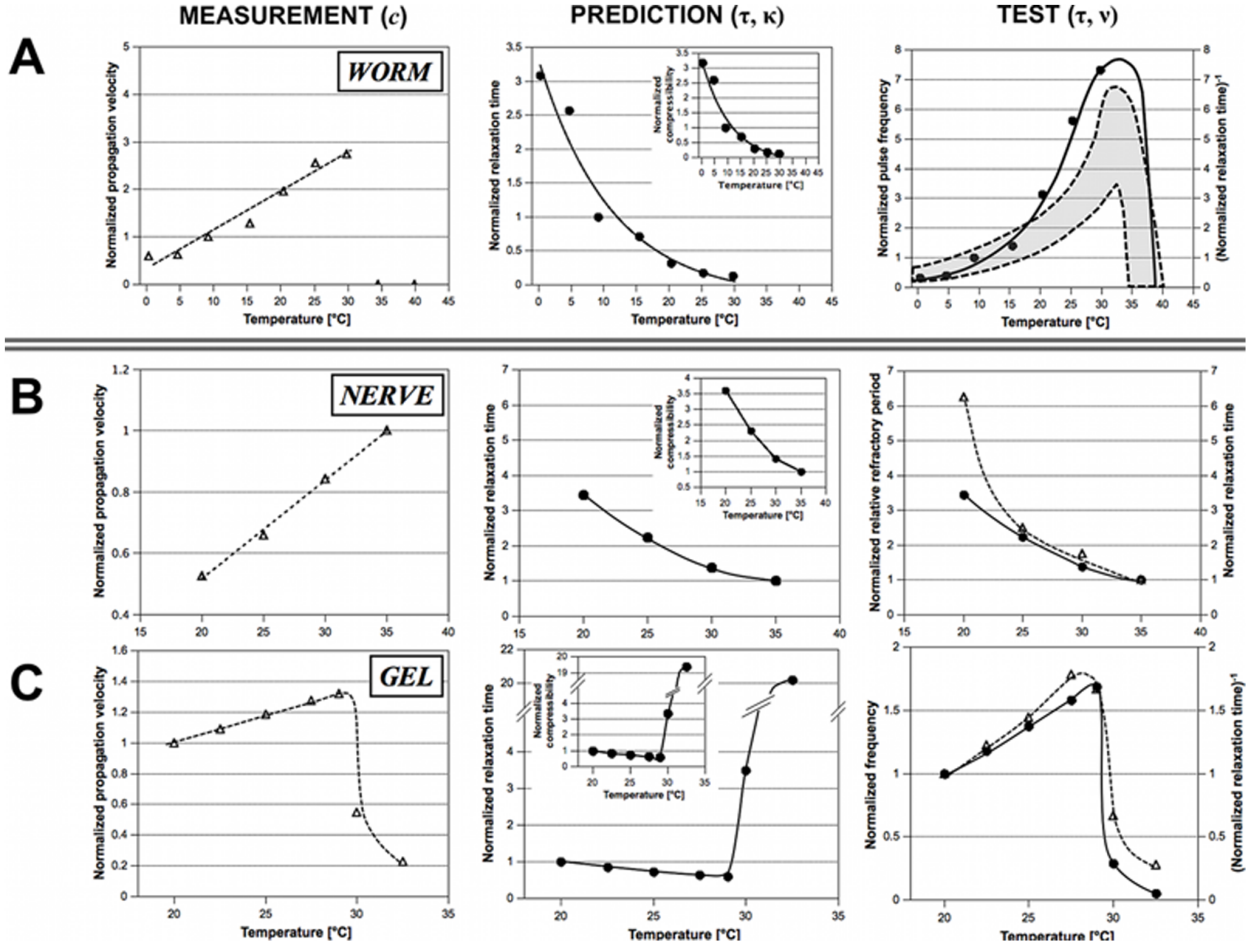
Thermodynamic approach: Experiments, predictions and comparisons. Based on experimentally obtained temperature-pulse velocity profiles *c* (left column), temperature-dependent compressibilities κ and relaxation times τ (middle column) were calculated for blood vessel pulsations in worms (A), action potentials in human nerves (B; data taken from Fig. 1 in [17]) and waves in gel rods (C; data taken from Fig. 3 and 4 in [16]). Comparison of predicted relaxation times τ and frequencies ν with their experimentally obtained counterparts (right column). Experimental data are always plotted as open triangles connected by broken lines while predictions are plotted as filled circles connected by solid lines.

To test if an acoustic theory and Eq. (1) adequately describe the velocity of pulses in nerve and muscle would require measurements of the state-dependent κ of the excitable medium (*e.g.* temperature dependence of κ; such experiments are presently underway in our lab). It is equally interesting, however, to use the experimentally observed temperature-velocity relationship for blood vessel pulsations in blackworms (Fig. 2) in order to make predictions about κ of the excitable medium. In fact, to determine a system’s compressibility from the velocity of a sound wave in it is a typical approach in material science. Essentially, low propagation velocities are expected to correspond to relatively higher compressibilities (“softer” system) and vice versa. From the data in Fig. 2 it is consequently predicted that the excitable medium in the dorsal blood vessel of the worm “hardens” with heating and that its material properties change profoundly in the vicinity of the heat block temperature Fig. 5A (middle, inset). The latter interpretation is supported by observations of discontinuous thermodynamic transitions in excitable gel rods [16], squid giant axons [31] and protoplasmic droplets of excitable cells [32] close to the typical heat block temperature.

#### Prediction of Relaxation Times

In a further step, the mechanical compressibilities estimated from the velocity-temperature relationship can be used to tentatively predict the timescales for relaxation processes in the system. By combining Einstein’s approach to thermodynamics (see Text S1) with an Onsager-type Ansatz, one can show that thermodynamic susceptibilities of a system (*e.g.* heat capacity, compressibility, etc.) are related to relaxation times τ. For lipid bilayer [33] and monolayer (see Text S1), where thermodynamic observables (area *A*, enthalpy *H*, charge *Q*, etc.) are coupled linearly, the expression becomes particularly simple:
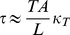
(2)where *T* is temperature, *A* the system’s surface area, *L* a phenomenological parameter and κ_T_ the isothermal compressibility. A similar relation between τ and the heat capacity *c_P_* has been derived [33]. If one assumes that the surface area as well as *L* of the excitable system does not change appreciably with temperature, relaxation times of the system can be predicted from temperature-dependent compressibilities. τ is expected to increase with increasing compressibility, which essentially means that softer systems relax slower. By combining Eq. (1) and Eq. (2) one also finds that 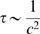 or expressed in terms of frequency ν (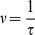):



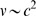
(3)Hence, propagation velocities are related to relaxation times and frequencies.

The thermodynamic relaxation times should for instance correlate to refractory periods after action potentials or spreading depression waves. In the case of systems with recurring auto-excitations (*e.g.* hearts) the reciprocal of the relaxation time is expected to represent the limiting (*i.e.* highest) frequency at which pulses can be generated.

#### Comparison with Experimental Data

The predictions of the presented thermodynamic concept can now be compared with experimental data. The temperature-velocity profile of blackworm blood vessel pulsations (Fig. 5A, left) served as a basis to calculate compressibilities and relaxation times (Fig. 5A, middle). The reciprocals of the relaxation times represent frequencies ν, which indeed agree well with the temperature-dependent pulse rates measured in blackworms (Fig. 5A, right). At least two interpretations are conceivable. First, two decoupled mechanisms determine the propagation velocity and pulse frequency in the dorsal vessel of *Lumbriculus*. The excitable medium in the blackworm’s blood vessel could be capable of transmitting pulses at higher frequencies than those observed experimentally. If this were the case, the temperature-frequency dependence in Fig. 3 would probably reflect the temperature dependence of an independent pacemaker mechanism. Second, the macroscopic thermodynamic properties of the excitable medium determine the propagation velocity *and* control the typical pulse frequency in the system. While this latter interpretation seems appealing by virtue of its integrative treatment of the system and a minimum of assumptions about a pacemaker mechanism, further experiments will have to test its validity.

To illustrate that the presented framework is by no means limited to a specific type of pulse or system, data on action potentials in human nerves [17] and on Belousov-Zhabotinsky reaction waves in gel rods [16] were extracted from the literature. The experimentally obtained temperature-pulse velocity profiles (Fig. 5 B and C, left) of these two systems were used to predict compressibilities and relaxation times (Fig. 5 B and C, middle). In the case of nerve action potentials, the calculated relaxation times correspond well with experimental data on refractory periods (Fig. 5B, right). Similarly, the temperature-dependent frequencies predicted for chemical waves in gels are in quite good agreement with the experimentally found ones (Fig. 5C, right). This overall consistency further underlines the general applicability and predictive power of the presented thermodynamic approach.

#### Comments and Outlook

A short comment concerning the classical interpretation of refractory periods after excitation waves in tissue should be made. Typically, it is assumed that the duration of the refractory period, for example ensuing a spreading depression wave, is determined by the timescales required by metabolic processes to re-establish ion gradients [34]. It should be emphasized that for the derivation of the relaxation times (Fig. 5) no assumptions about metabolic reactions or equilibration processes outside of the excitable medium had to be made. Thus, we believe that it would be worthwhile to scrutinize if it is indeed necessary to invoke metabolic reactions or other mechanisms to explain refractory periods in biological systems.

Finally, it is important to point out key questions that have been left untouched herein. *(i)* Measurements of the temperature-dependent compressibility of excitable media will allow to directly challenge the presented concept (such experiments are currently underway in our lab). *(ii)* While the temperature of the preparation (worm, nerve, etc.) is rather easily “clamped” in experiments, the other thermodynamic state variables of the cell or tissue are free to vary (*e.g.* lateral pressure of the cell membrane, pH close to the cell membrane, etc.). This has to be kept in mind when interpreting phenomena in the system. *(iii)* It has to be emphasized that the expression for linear sound (Eq. (1)) can only serve as a reasonable first order approximation. Indeed, we do not believe that the observed pulses are linear phenomena over the entire temperature range. The square root dependence between *c* and κ, however, is a fairly good approximation as longs as the variations of κ with density are rather moderate compared to κ [35]. *(iiii)* Dispersion and collision phenomena, which are readily observed in nerves [36], nervous tissue [14] and non-living excitable media [37], cannot be described in the linear regime. The characteristic annihilation of pulses is certainly a main challenge since neither a linear sound nor a soliton model [38] is capable of explaining it. However, the existence of a transition (state-change) during pulse propagation as proposed in the soliton model appears to open new doors to tackle this problem [3].

### Conclusions

Experimental data on the effect of temperature on blood vessel contraction velocity and frequency in *Lumbriculus variegatus* were presented. These results were interpreted in the framework of a thermodynamic theory. Testable predictions about the temperature dependence of the excitable medium’s mechanical material properties were made. Based on these predictions, temperature-dependent relaxation timescales were derived which correlate with experimentally obtained data from blackworms, nerves and gels.

When trying to find a physical explanation for phenomena of such remarkable generality, one has to carefully consider the assumptions made. In this spirit, we believe that the universal character of thermodynamics combined with as few specific assumptions as possible is the most promising approach to provide a robust and testable explanation.

## Supporting Information

Text S1 Einstein’s approach to thermodynamics – Derivation of Eq. (2).
(PDF)
