# Supplementary material for: On the Temperature Behavior of Pulse Propagation and Relaxation in Worms, Nerves and Gels
Source: PLoS One. 2013 Jun 21;8(6):e66773. doi: 10.1371/journal.pone.0066773 (PMC3689679; doi:10.1371/journal.pone.0066773)
Supplement: Text S1 — Einstein’s approach to thermodynamics – Derivation of Eq. (2). (PDF) [file pone.0066773.s001.pdf]

Text S1

Einstein's approach to thermodynamics. In the following we present a brief outline of the derivation of Eq. (2). For detail please refer to the work cited below.

In 1910 Einstein published a paper in which he presented what has been later called (for instance by E. Schrödinger and A. Sommerfeld) „Einstein's reversion of thermodynamics" [39]. Einstein calls it „meaningless" to use Boltzmann's principle by starting from a molecular theory. Instead, Boltzmann's principle – which he believed to be correct - has to be applied in the reversed order: One starts from the established thermodynamic phenomenology of the system and then uses it to derive the system's fluctuations. This way, microscopic consequences can be directly derived from macroscopic observations and the second law of thermodynamics without molecular or statistical mechanics theory. The formalism of Einstein starts from a Taylor expansion of the entropy *S* (for simplicity here in one variable; more details can be found in [39–42]:

$$\boldsymbol{S} \approx \boldsymbol{S}_0 + \frac{\partial \boldsymbol{S}}{\partial \boldsymbol{n}}(\boldsymbol{\delta n}) + \frac{1}{2}\left(\frac{\partial^2 \boldsymbol{S}}{\partial \boldsymbol{n}^2}\right)(\boldsymbol{\delta n})^2 \qquad (1)$$

where S_0 is the entropy maximum and *n* a thermodynamic variable. Combined with Boltzmann's principle (k_b Boltzmann constant) $P = \exp(S/k_B)$ and near equilibrium, where $\partial S/\partial n \approx 0$, this leads to a Gaussian probability distribution for the variable

$$\boldsymbol{P}(\boldsymbol{n}) \approx \boldsymbol{C}_0 \exp\left[-\frac{\boldsymbol{\beta}}{2\boldsymbol{k}_B}\boldsymbol{n}^2\right] \qquad (2)$$

The standard deviation (thermodynamic fluctuations) of *P(n)* turns out to be directly proportional to the susceptibility of the system, *e.g.,* in the case where *n* represents the area *A* of a system, to the lateral isothermal compressibility:

$$\left\langle (\delta A)^2 \right\rangle = -k_B \left(\frac{\partial^2 S}{\partial A^2}\right)^{-1} = k_B T \cdot A \cdot \kappa_T \qquad (3)$$

This concept has been applied to lipid bilayers [40,43–45] as well as to lipid monolayers more recently [46].

Heimburg has combined Einstein's approach with an Onsager type Ansatz to find a relation between heat capacity and relaxation time [33], which has been applied successfully to lipid membranes [43]. Similarly, one can demonstrate that based on a relation between area flux and corresponding thermodynamic force

$$\frac{\partial A'}{\partial t} = L\frac{\partial S}{\partial A'} \tag{4}$$

combined with a Taylor expansion of the first derivative

$$\frac{\partial S}{\partial A} \approx \frac{\partial^2 S}{\partial A^2} \cdot A' \tag{5}$$

and Eq. (S3), an expression between relaxation time τ and compressibility κ is derived

$$\frac{1}{\tau} \approx -L\left(\frac{\partial^2 S}{\partial A^2}\right)_{A_0} = -\frac{L}{T \cdot A \cdot \kappa_T} \tag{6}$$

which is identical to Eq. (2) in the manuscript.

Additional references

39. Einstein A (1910) Theory of the opalescence of homogenous fluids and liquid mixtures near the critical state. Annalen der Physik 33: 1275–1298.

40. Kaufmann K, Hanke W, Corcia A (1989) Ion channel fluctuations in pure lipid bilayer membranes: Control by voltage. Caruaru, Brazil.

41. Einstein A (1909) On the present status of the radiation problem. Physikalische Zeitschrift 10: 185–193.

42. Einstein A (1913) Zum gegenwärtigen Stande des Problems der spezifischen Wärme. Abhandlungen Bunsengesellschaft 3: 330–364.

43. Wunderlich B, Leirer C, Idzko A-L, Keyser UF, Wixforth A, et al. (2009) Phase-state dependent current fluctuations in pure lipid membranes. Biophysical Journal 96: 4592–4597.

44. Blicher A, Wodzinska K, Fidorra M, Winterhalter M, Heimburg T (2009) The temperature dependence of lipid membrane permeability, its quantized nature, and the influence of anesthetics. Biophysical Journal 96: 4581–4591.

45. Nagle J, Scott Jr H (1978) Lateral compressibility of lipid mono-and bilayers. Theory of membrane permeability. Biochimica et Biophysica Acta -Biomembranes 513: 236–243.

46. Steppich D, Griesbauer J, Frommelt T, Appelt W, Wixforth A, et al. (2010) Thermomechanic-electrical coupling in phospholipid monolayers near the critical point. Physical Review E 81: 061123.
